# Timing of Gestational Weight Gain on Fetal Growth and Infant Size at Birth in Vietnam

**DOI:** 10.1371/journal.pone.0170192

**Published:** 2017-01-23

**Authors:** Melissa F. Young, Phuong Hong Nguyen, O. Yaw Addo, Hoa Pham, Son Nguyen, Reynaldo Martorell, Usha Ramakrishnan

**Affiliations:** 1 The Hubert Department of Global Health Emory University, Atlanta, GA, United States of America; 2 Thai Nguyen University of Pharmacy and Medicine, Thai Nguyen, Vietnam; 3 International Food Policy Research Institute, Washington DC, United States of America; Shoklo Malaria Research Unit, THAILAND

## Abstract

**Objective:**

To examine the importance of timing of gestational weight gain during three time periods: 1: ≤ 20 weeks gestation), 2: 21–29 weeks) and 3: ≥ 30 weeks) on fetal growth and infant birth size.

**Methods:**

Study uses secondary data from the PRECONCEPT randomized controlled trial in Thai Nguyen province, Vietnam (n = 1436). Prospective data were collected on women starting pre-pregnancy through delivery. Maternal conditional weight gain (CWG) was defined as window-specific weight gains, uncorrelated with pre-pregnancy body mass index and all prior body weights. Fetal biometry, was assessed by ultrasound measurements of head and abdomen circumferences, biparietal diameter, and femoral length throughout pregnancy. Birth size outcomes included weight and length, and head, abdomen and mid upper arm circumferences as well as small for gestational age (SGA). Adjusted generalized linear and logistic models were used to examine associations.

**Results:**

Overall, three-quarters of women gained below the Institute of Medicine guidelines, and these women were 2.5 times more likely to give birth to a SGA infant. Maternal CWG in the first window (≤ 20 weeks), followed by 21–29 weeks, had the greatest association on all parameters of fetal growth (except abdomen circumference) and infant size at birth. For birth weight, a 1 SD increase CWG in the first 20 weeks had 3 times the influence compared to later CWG (≥ 30 weeks) (111 g vs. 39 g) and was associated with a 43% reduction in SGA risk (OR (95% CI): 0.57 (0.46–0.70).

**Conclusion:**

There is a need to target women before or early in pregnancy to ensure adequate nutrition to maximize impact on fetal growth and birth size.

**Trial Registration:**

ClinicalTrials.gov, NCT01665378

## Introduction

Maternal undernutrition is a key determinant of poor fetal growth, low birthweight, and increased infant morbidity and mortality [1]. Several studies have examined the association of total gestational weight gain on adverse birth outcomes [2], however the evidence on the relative importance of timing of gestational weight gain remains inconclusive [3–13]. While the majority of weight gain occurs in the second and third trimesters, it is unclear whether small gains in early pregnancy may be as important as larger gains later on. Few studies have high quality prospective data from preconception to delivery to accurately capture early weight gain to address this question. There is paucity of evidence on when maternal weight gain matters most and how this corresponds with fetal growth in-utero and birth outcomes (many studies have focused solely on birthweight [2]). A potential reason for the mixed results are the inherent methodological limitations of modeling highly correlated weight measures across pregnancy. To assess trimester specific influences on newborn outcomes advanced statistical methods are needed to generate independent and window-specific measures of maternal weight gain.

Using prospective data starting pre-pregnancy through delivery from the PRECONCEPT randomized controlled trial in Thai Nguyen, Vietnam, we have previously demonstrated similar and independent effects of maternal nutrition before and during pregnancy on birth outcomes [14]. In the current study, our objective is to examine the relative importance of timing of gestational weight gain during pregnancy on fetal growth and birth outcomes.

## Materials and Methods

### Data sources and study population

**Fig 1:**
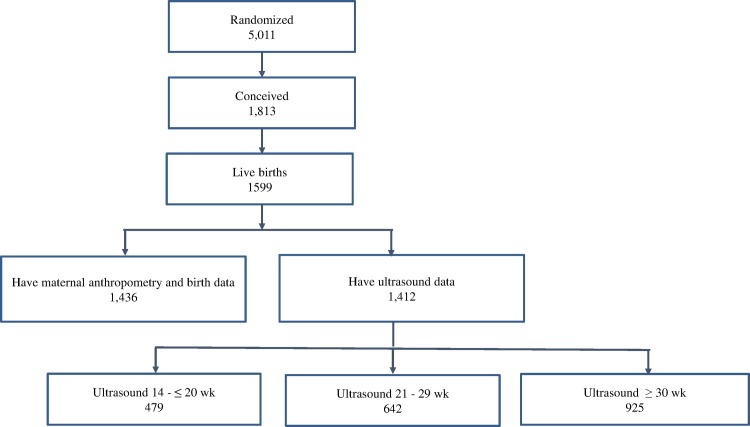
Flow diagram of participant progress throughout the study.

This study uses secondary data obtained from a randomized controlled trial, PRECONCEPT study, evaluating the effects of preconception micronutrient supplementation on maternal and child health outcomes [15]. The parent study was approved by the Ethical Committee of Institute of Social and Medicine Studies in Vietnam and Emory University's Institutional Review Board, Atlanta, Georgia, USA (ClinicalTrials.gov, NCT01665378) Written informed consent was obtained from all study participants in accordance with approved ethics committes. This trial was conducted in 20 rural communes in Thai Nguyen province, in North Vietnam. Between October 2011- May 2012, a total of 5011 women of reproductive age were randomly allocated to receive weekly supplements containing either multiple micronutrients, iron-folic acid or folic acid only. These women were followed prospectively to monitor pregnancies and birth outcomes. A total of 1,813 women conceived between 2012–2014 and 1,599 had live births. The current analysis includes a sub-set of 1436 women who delivered singleton live infants with available data on maternal anthropometry before and during pregnancy and offspring birth size (Fig 1). Of these women 1,412 women have ultrasound data contributing 2,200 ultrasound measurments; 738 women had 2–3 ultrasound measurements over the course of pregnancy and 675 women had one measure.

### Outcome measurements

#### Fetal size

Fetal measurements including head circumference (HC), biparietal diameter (BPD), abdominal (AC) and femur length (FL) were obtained during three time periods 1:14 - ≤ 20 weeks, 2: 21–29 weeks) and 3: ≥ 30 weeks) pregnancy at commune health clinics by trained obstetricians, using real-time ultrasound on a portable machine (Prosound 2, Hitachi Aloka, Japan). Details of the ultrasound examination techniques that were used have been described elsewhere [16]. Briefly, HC were measured based on ellipse fitting on the outer border of the skull using standard techniques [17]. BPD was measured from the outer edge of the proximal parietal bone to the outer edge of the distal parietal bone [17]. Femur length was measured according to the method of O’Brien and Queenan [18]. Abdominal circumference was measured either directly by tracing around the perimeter (for second trimester of pregnancy) or indirectly using the formula π (d1+ d2)/2 (for the third trimester of pregnancy), where d1 and d2 are the transverse and antero- posterior diameters [19]. Duplicate measures were obtained from separate scans and the averages were used for analyses. High levels of quality control were in place for the study including: careful and detailed in baseline training, refresher training every 3 months as well as random supervisor check-up throughout the course of the study.

#### Newborn measurements

Newborn anthropometric measurements, including weight and length, and head, abdomen and mid upper arm circumferences, were measured within 7 days of delivery following standardized techniques [20]. Naked weight was measured using a UNICEF Beam type scale. Recumbent length was measured using a wooden measuring board and circumferences were measured with non-stretchable UNICEF tape. The average of duplicate anthropometric measurements were used for analyses.

Gestational age (GA) was assessed based on prospective data collection on last mentral period (LMP) to estimate date of conception and on date of delivery; a modified LMP method superior to maternal recall of LMP during pregnancy or delivery [15]. Health workers visited the homes of women every two weeks after preconception enrollment and were asked about their menses. If women reported that their LMP was >5 weeks, urine pregnancy tests were done at a local Commune Health Center. Thus, we were able to obtain highly accurate estimates of GA as calculated by the number of days between LMP and delivery. Previous research from our team have also established a high correlation between GA as estimated from prospectively-collected LMP and ultrasound measurements [21, 22]. Small for gestational age (SGA) was defined as a birthweight below the 10th percentile for GA based on the international newborn standards from the INTERGROWTH-21st Project [23].

### Predictor variables

Maternal pre-pregnancy weight and height were measured at enrollment by trained workers using standard procedures [24]. Pre-pregnancy body mass index (PP-BMI) was calculated as weight/height^2^ (kg/ m^2^). Underweight was defined as BMI <18.5 kg/ m^2^ and overweight was defined >23 kg/ m^2^. [25] Gestational weight gain was calculated from maternal weight measured at delivery and pre-pregnancy weight. We compared the adequacy of gestational weight gain in relation to Institute of Medicine (IOM) recommendations to define those above or below IOM recommendation [2].

The conditional gestation weight (CWG) variable was developed to produce uncorrelated gestation weight gain for specific windows of pregnancy. CWG is a derived variable and computed as the studentized residuals in linear models in which the outcome variable is maternal weight at any given time point during pregnancy; and the predictor variables are her own pre-conception BMI and any prior pregnancy weights. Conditional gestational weight gain were computed for 3 windows: 1: ≤ 20 weeks, 2: 21–29 weeks and 3: ≥ 30 weeks (these are the same windows as described for fetal growth with the exception of window 1; for fetal growth the earliest ultrasound is 14 weeks and for maternal weight gain the window begins at preconception enrollment). Each conditional gestation weight measurement can be interpreted as weight deviation from the expectant mothers own projected weight trajectory, and thus is an indicator of relative speed of weight gain/loss within an interval of pregnancy, independent of her weight at a previous time point(s). Such methods have been used by the COHORTS collaboration to study relationships between growth from birth to adulthood and adult human capital and health [26]. Conditional variables are necessary in examining the time-specific influence of maternal weight gain changes on birth outcomes because measurements at each time point during pregnancy are related to all prior ones and could be placed a single linear regression without any inflated variances.

### Confounders

Socio-demographic characteristics include maternal age, education, ethnicity and social economic status (SES). The SES index was constructed using principal components analysis that accounted for a variety of variables such as house and land ownership, household assets, and access to services [27, 28], and used to categorize subjects into quintiles where higher scores indicate wealthier participants. We also controlled for the treatment group (iron and folic acid, multiple micronutrients or folic acid alone) of the PRECONCEPT randomized trial, child gender, and time from enrolment to conception.

### Statistical analysis

Descriptive analyses were used to report socio-demographic characteristics of the population. Normality of the continuous outcome variables was assessed using the Kolmogorov-Smirnov test. Ultrasound measures of HC, BPD, AC, and FL were also converted to Z scores using the reference values from the INTERGROWTH-21st Project [29]. Since each CWG measure is independent of pregnancy BMI and weight gain in any prior window, we included multiple measures of maternal weight in the same model to examine the independent effect of weight gain in each window. To examine the association of maternal weight gain on fetal growth, three separate models were built by regressing window specific maternal CWG (as the key predictor) against fetal growth outcomes (examined separately for biparietal diameter, head and abdominal circumferences, and femur length, all in SD units while controlling for potential confounding factors). Model 1 examined relations between maternal CWG in the first pregnancy window and fetal growth from ultrasounds 14 - ≤ 20 weeks. Similarly model 2 looked at the first two windows of weight gain and ultrasounds for 21–29 weeks, and the final model looked at all 3 windows of weight gain and the final ultrasounds for ≥ 30 weeks. For example to predict late fetal growth, head circumference z-score = CWG ≤ 20 weeks + CWG 21–29 weeks + CWG ≥ 30 weeks + maternal prepregnancy BMI + Covariates (ethnicity, maternal age, education, treatment group, child gender, household SES, time from enrolment to conception and repeated measure). Likewise, we examined the relative importance for timing of CWG on birth outcomes using generalized linear models, adjusting for potential confounding factors at household, maternal and child level (described above). Statistical tests were 2-tailed and differences were considered significant at P <0.05. SAS software, version 9.3 was used for statistical analysis.

## Results

Approximately one-third of women entered pregnancy underweight with a mean pre-pregnancy weight of 45.8 kg. The average gestational weight gain was 10 kg, with nearly three-quarters of women gaining below the IOM recommended guidelines for weight gain during pregnancy (Table 1). Mean birth weight was 3050g and 15.7% of infants were born small for gestational age. Characteristics of those included in analysis are similar to the primary cohort of women at enrollment (results not shown).

**Table 1:** Maternal and newborn characteristics (n = 1436)^1^.

Characteristic	Mean ± SD or %
**Maternal Indicators**	
Age at baseline (y)	25.8 ± 4.3
Primiparous (%)	5.5
Pre-pregnancy Weight (kg)	45.8 ± 5.4
Height (m)	152.7 ± 5.1
Pre-pregnancy BMI (kg/m2)	19.6 ± 2.0
BMI < 18.5 (%)	31.1
BMI > 23 (%)	5.9
Gestational Weight gain (kg)	10.0 ± 4.0
Gained below IOM recommendation (%)	73.4
Gained at IOM recommendation (%)	23.5
Gained above IOM recommendation (%)	5.1
**Newborn Indicators**	
Female (%)	48.2
Birth weight (g)	3050 ± 396.6
Birth length (cm)	49.0 ± 2.8
Head circumference (cm)	32.3 ± 2.5
MUAC (cm)	11.0 ± 1.7
Abdominal circumference (cm)	31.1 ± 2.7
Preterm birth (%)	9.7
SGA (%)	15.7

^1^BMI, body mass index; IOM, Institute of Medicine; MUAC, mid-upper-arm circumference; SGA, small for gestational age based on INTERGROWTH-21^st^ project.

Associations between maternal CWG during the different time periods and various measures of fetal ultrasound growth (HC, BPD, AC and FL z-scores) throughout pregnancy are shown in Table 2. The first time period of maternal weight gain (≤ 20 weeks) was significantly associated with fetal ultrasound growth measures during early (BPD), mid and late pregnancy (HC, BPD, FL). For example, a one standard deviation increase in maternal CWG in the first 20 weeks of pregnancy was associated with a 0.17 increase in BPD z-score as detected by ultrasound measures during the 14 - ≤ 20 week visit (Table 2A). Maternal weight gain in the first 20 weeks of pregnancy compared to weight gain in the second window (21–29 weeks) had nearly twice the influence on late fetal growth (Table 2C) for HC (a 1 SD increase in CWG was associated with a 0.26 and 0.14 increase in HC z-score, respectively), BPD (0.29 vs. 0.15), and FL (0.23 vs. 0.17) with the exception of AC in which CWG during 21–29 weeks had the greatest association. Pregnancy weight gain during the last window (≥ 30 weeks) was not significantly associated with fetal growth.

**Table 2:** Associations between maternal conditional weight gain and fetal growth during early (14 - ≤ 20 weeks), mid (21–29 weeks) and late (≥ 30 weeks) pregnancy^1^.

	Head circumference Z-score	Biparietal diameter Z-score	Abdomen circumference Z-score	Femoral Length Z-score
Maternal Conditional Weight Gain; Z-scores	β (95% CI)	β (95% CI)	β (95% CI)	β (95% CI)
**A. Model 1: pregnancy fetal growth during 14 - ≤ 20 weeks; n = 479**				
CWG ≤ 20 weeks	0.11 (-0.06,0.27)	0.17* (0.00,0.34)	0.1 (-0.09,0.29)	0.09 (-0.08,0.26)
**B. Model 2: pregnancy fetal growth during 21–29 weeks; n = 642**				
CWG ≤ 20 weeks	0.24** (0.09,0.38)	0.21** (0.07,0.36)	0.13 (-0.03,0.30)	0.29*** (0.15,0.44)
CWG 21–29 weeks	0.17* (0.02,0.32)	0.12 (-0.03,0.26)	0.20* (0.03,0.37)	0.17* (0.03,0.32)
**C. Model 3: pregnancy fetal growth during ≥ 30 weeks; n = 925**				
CWG ≤ 20 weeks	0.26***(0.17,0.36)	0.29*** (0.20,0.38)	0.07 (-0.03,0.17)	0.23*** (0.12,0.33)
CWG 21–29 weeks	0.14** (0.04,0.24)	0.15** (0.06,0.24)	0.25*** (0.15,0.36)	0.17** (0.05,0.28)
CWG ≥ 30 weeks	0.01 (-0.09,0.10)	0.04 (-0.05,0.13)	0.04 (-0.06,0.14)	-0.04 (-0.15,0.06)

CI, confidence interval; CWG, conditional weight gain

^1^ Significance level: *p<0.05 **p<0.01 ***p<0.001; Each model was run separately for each fetal growth outcome and for each time period (A. 14 - ≤ 20 weeks, B. 21–29 weeks and C. ≥ 30 weeks). Models adjusted for maternal prepregnancy BMI, ethnicity, maternal age, education, treatment group, child gender, household SES, time from enrolment to conception and repeated measure; Conditional gestational weight gain during pregnancy: each window is independent of pre-pregnancy BMI and independent of weight gain in any prior window. Units are in standardized z-scores to allow for relative comparisons of a 1 SD average increase in weight gain for each window.

Similarly, maternal CWG during the first period had the greatest association on all infant outcomes at birth (Table 3). A 1 standard deviation (SD) increased in CWG first 20 weeks of pregnancy was associated with a 111 g increase in birth weight, 0.5 cm increase in length, 0.4 cm increase in head circumference, 0.2 cm increase in MUAC and a 0.5 cm increase in abdomen circumference. Maternal CWG during 21–29 weeks had the second largest association while CWG in the last window of pregnancy (≥ 30 weeks) had the weakest association with birth outcomes. For infant birth weight, a 1 SD increase in weight gain earlier in pregnancy (≤ 20 weeks) had nearly three times the influence compared to the same weight gain later (≥ 30 weeks) in pregnancy (111 vs. 39 grams).

**Table 3:** Conditional weight gain during pregnancy and infant birth size^1^ (n = 1436).

	Weight (g)	Length (cm)	Head (cm)	MUAC (cm)	Abdomen (cm)
Maternal variables; Z-score	β (95% CI)	β (95% CI)	β (95% CI)	β (95% CI)	β (95% CI)
PP BMI	88.65**(64.5, 112.8)	0.28 (0.08, 0.49)	0.29**(0.12, 0.46)	0.07(-0.03, 0.17)	0.24*(0.06, 0.42)
≤ 20 weeks CWG	111.40**(88.1, 134.7)	0.45**(0.25, 0.64)	0.38**(0.22, 0.54)	0.20**(0.10, 0.30)	0.54**(0.37, 0.72)
21–29 weeks, CWG	71.17**(47.0, 95.3)	0.30*(0.09, 0.50)	0.25*(0.08, 0.41)	0.13*(0.03, 0.23)	0.26*(0.07, 0.43)
≥ 30 weeks, CWG	38.94*(14.7, 63.1)	0.29*(0.09, 0.49)	0.09(-0.07, 0.26)	0.07(-0.03, 0.17)	-0.04(-0.22, 0.147)

^1^ Significance level: *p<0.05 **p<0.01; Model adjusted for maternal prepregnancy BMI z-score, ethnicity, maternal age, education, treatment group, child gender, household SES, time from enrolment to conception and repeated measure; Conditional gestational weight gain during pregnancy: each window is independent of pre-pregnancy (PP) BMI and of weight gain in any prior window. Units are in standardized z-scores to allow for relative comparisons of a 1 SD average increase in weight gain for each window. A 1 SD weight gain per woman for each period (≤ 20 wk, 21–29 wk and ≥ 30 wk) is 2.4 kg, 2.3 kg and 2.9 kg respectively. MUAC, mid-upper-arm circumference; CI, confidence interval; BMI, body mass index; CWG, conditional weight gain.

The influence of maternal weight gain during pregnancy on risk for giving birth to a SGA infant is presented in Table 4. One standard deviation increase in CWG in the first 20 weeks of pregnancy was associated with a 48% reduction in risk of SGA and had nearly twice the influence compared to later pregnancy weight gain, ≥ 30 weeks (odds ratios 0.52 vs. 0.78). In terms of total gestational weight gain, women who gained below the IOM recommended guidelines were 2.5 times more likely to give birth to a SGA infant as compared to women who gained within the IOM recommendations

**Table 4:** Weight gain during pregnancy and risk for SGA^1^.

	Odds Ratio (95% CI)
**Conditional weight gain; Z-score (n = 1436)**	
≤ 20 weeks	0.52** (0.42, 0.66)
21–29 weeks	0.76** (0.61, 0.95)
≥ 30 weeks	0.78** (0.63, 0.97)
**Total gestational weight gain**	
Gained below IOM recommendations **(n = 1054, 73%)**	2.54** (1.6, 4.0)

CI, confidence interval; IOM, Institute of Medicine.

^1^Significance level: *p<0.05 **p<0.01 ***p<0.001. In comparison to 2009 Institute of Medicine weight gain recommendations according to prepregnancy BMI (reference group: women who gained within guidelines).; CWG: each window is independent of pre-pregnancy BMI and independent of weight gain in any prior window; All models adjusted for pre-pregnancy BMI, gestational age, sex of child, age of mother, ethnicity, education, SES, treatment group, time (first prenatal-baseline), ethnicity.

## Discussion

Novel data on the relative importance of timing of weight gain during pregnancy are presented that support the need to target women before and early in pregnancy to improve birth outcomes. This paper expands on our previous research demonstrating a similar and independent impact of maternal nutrition before (pre-pregnancy weight) and during (total gestational weight gain) pregnancy [14] and provides evidence that the timing of weight gain also matters. By using advanced statistical methods the importance of conditional weight gain during three time periods (≤ 20 wk, 21–29 wk and ≥ 30 wk) pregnancy was examined. Weight gain in the first 20 weeks of pregnancy was a particularly important window for fetal growth and infant birth outcomes; having 2–3 times the influence as weight gained later in pregnancy. In our population, three quarters of women gained below the IOM recommendations and these women were 2.5 times more likely to give birth to a SGA baby. This research is consistent with prior research confirming the importance of maternal nutrition during pregnancy [2, 14, 30].

The recent release of international standards for fetal growth (INTERGROWTH -21^st^ [23]) allows for examining patterns of fetal growth across pregnancy [16, 23]. Previous research in our population has illustrated that fetal growth failure begins early in pregnancy and continues throughout delivery, which places infants at risk for SGA [16]. In this study, we presented that early CWG within first 20 weeks is an important predictor for fetal growth in utero and preventing fetal growth restriction. All fetal ultrasounds measures, with the exception of abdominal circumference, were most strongly associated with CWG during the first 20 weeks of pregnancy, compared to 21–29 weeks or weight gain after 30 weeks. We are not certain why reationships with abdominal circumference differ. It is possible that differences in ultrasound measurement technique using the Chitty et al., approach compared to the Villar et al approach may have contributed to these difference. [19, 23] However, potential bias is minized since we had trained obstetricians who were able to accurately measure AC (i.e. fit into the screen). Further research is needed to understand the complex underlying mechanisms of early fetal programming and the maternal/placental environment that may account for the importance of early nutrition.

In addition, CWG during the first time period (≤ 20 weeks) had the largest association on a range of infant birth outcomes as well. For example, a 1 SD deviation (2.4 kg) increase in maternal CWG (≤ 20 weeks) was associated with 111.4 g increase in birth weight. This is in line with prior US research, where one Kg of weight gain in the first trimester was associated with a 31 g increase in birthweight, in the second trimester the same weight gain was associated with a 26 g increase and third trimester was non-significant [5]. Research on the relative influence on gestional weight gain however remains mixed and some studies have suggested the second trimester is the most influential [3, 7]. In addition to inherent statistical challenges of examining correlated measurements of weight gain, the association between maternal nutrition and birth outcomes is complex and is influenced by many biological, socioecomonic and demographic factors [31], requiring examination to understand the differences among populations.

There are several strengths and limitations to our study. First, our large prospective cohort of women enrolled before conception allowed for accurate measures of gestational age and enabled us to assess the influence of maternal weight gain during preconception to 20 weeks. This critical early time period is typically missed as many women seek prenatal care after 20 weeks and pre-pregnancy weight is based on recall. In future research it would be interesting to further divide the first period to understand the role of weight gain/loss in first trimester alone. Another key strength is the availability of repeated fetal ultrasound data throughout pregnancy to enable the examination of fetal growth compared to international standards. However, we did not have sufficient data to examine fetal growth in the first trimester and the earliest fetal ultrasounds were obtained at 14 weeks. Finally, while many studies focus solely on birthweight, our study examined multiple measures of infant anthropometry at birth. The primary limitation of this study is the relatively homogenous population. The influence of timing of weight gain on LGA, excess adiposity or C-section risk were not able to be assessed given the overall low rates of overweight (BMI >23: 6%) and excessive weight gain (above IOM recommendation: 5%). While in our population insufficient weight gain and risk for SGA was the primary concern; the growing obesity epidemic and dietary transition in many developing countries cautions over weight gain is likewise important. In a study by Davenport et al., women who gained excessively in the first half of pregnancy were more likely to give birth to infants with elevated body fat [32]. In addition, in a Greece cohort weight gain in the first trimester was associated with cardiometabolic risk and child obesity [33]. Thus, balanced interventions that target women early in pregnancy to gain appropriate weight given their pre-pregnancy BMI are needed.

In conclusion, our results demonstrate that weight gain during the first 20 weeks pregnancy has the greatest relative influence on fetal growth and risk of SGA. This research has implications for programs on the need to target nutrition counselling and support to women early in pregnancy to optimize infant birth outcomes. Since many women do not seek prenatal care until mid pregnancy (~20 weeks) in many resource poor settings alternative strategies for reaching women before and in early in pregnancy may need to be considered and receive support.

## Supporting Information

S1 File Vietnam Dataset_anonymized.xlsx Vietnam datasest. (XLSX)
